# Supplementary material for: Computational mechanisms of distributed value representations and mixed learning strategies
Source: Nat Commun. 2021 Dec 10;12:7191. doi: 10.1038/s41467-021-27413-2 (PMC8664930; doi:10.1038/s41467-021-27413-2)
Supplement: Supplementary file 1 — Supplementary Information [file 41467_2021_27413_MOESM1_ESM.pdf]

Supplementary Information

Computational mechanisms of distributed value representations and mixed learning strategies

Shiva Farashahi* and Alireza Soltani*

**Corresponding authors:* AS, Department of Psychological and Brain Sciences, Dartmouth College, Hanover NH 03755, soltani@dartmouth.edu; SF, Flatiron Institute, Simons Foundation, New York NY 10010, sfarashahi@flatironinstitue.org

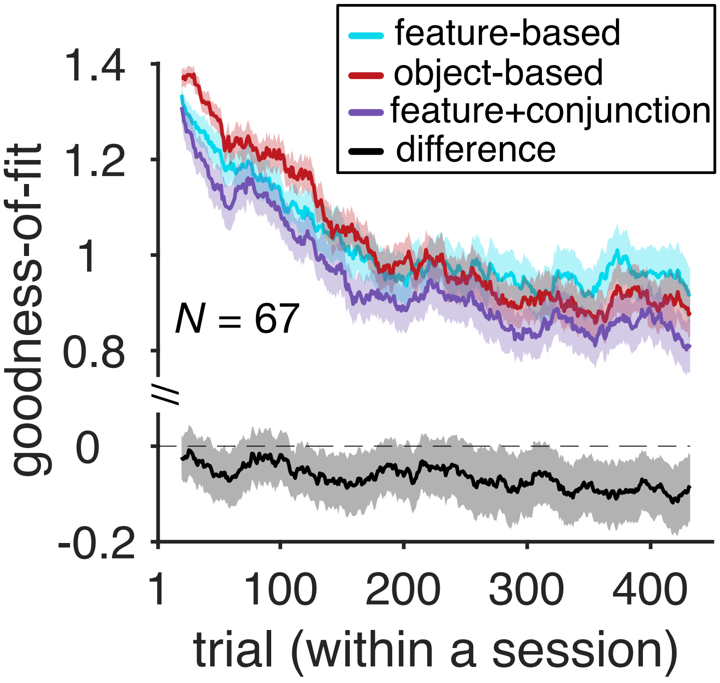

Supplementary Fig. 1. Evidence for adoption of mixed feature- and conjunction-based learning. Plotted is the goodness-of-fit based on the average BIC per trial, BIC_p, for the feature-based model, object-based model, and the best mixed feature- and conjunction-based ($F+C_1$) model. The smaller value corresponds to a better fit. The black curve shows the difference between the goodness-of-fit for the $F+C_1$ model and the feature-based model. The shaded areas indicate +/- s.e.m. Source data are provided as a Source Data file.

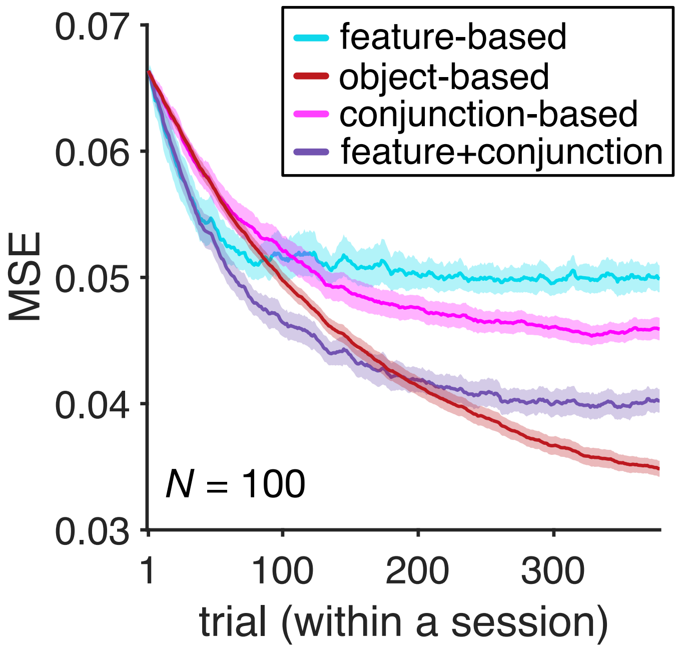

Supplementary Fig. 2. Time course of error in estimation of reward probabilities by different models. Plot shows the mean squared error (MSE) in the estimation of reward probabilities used in our learning task based on reinforcement learning models with decay and different learning strategies. The learning rates (α_{rew}, α_{unr}) were set to 0.05, and the decay rate as d = 0.005. A feature-based learner and a mixed feature- and conjunction-based learner (F+C_1) exhibits a lower MSE at the beginning of the experiment, whereas an object-based learner provides more accurate estimates later in the experiment. The shaded areas indicate +/- s.e.m. Source data are provided as a Source Data file.

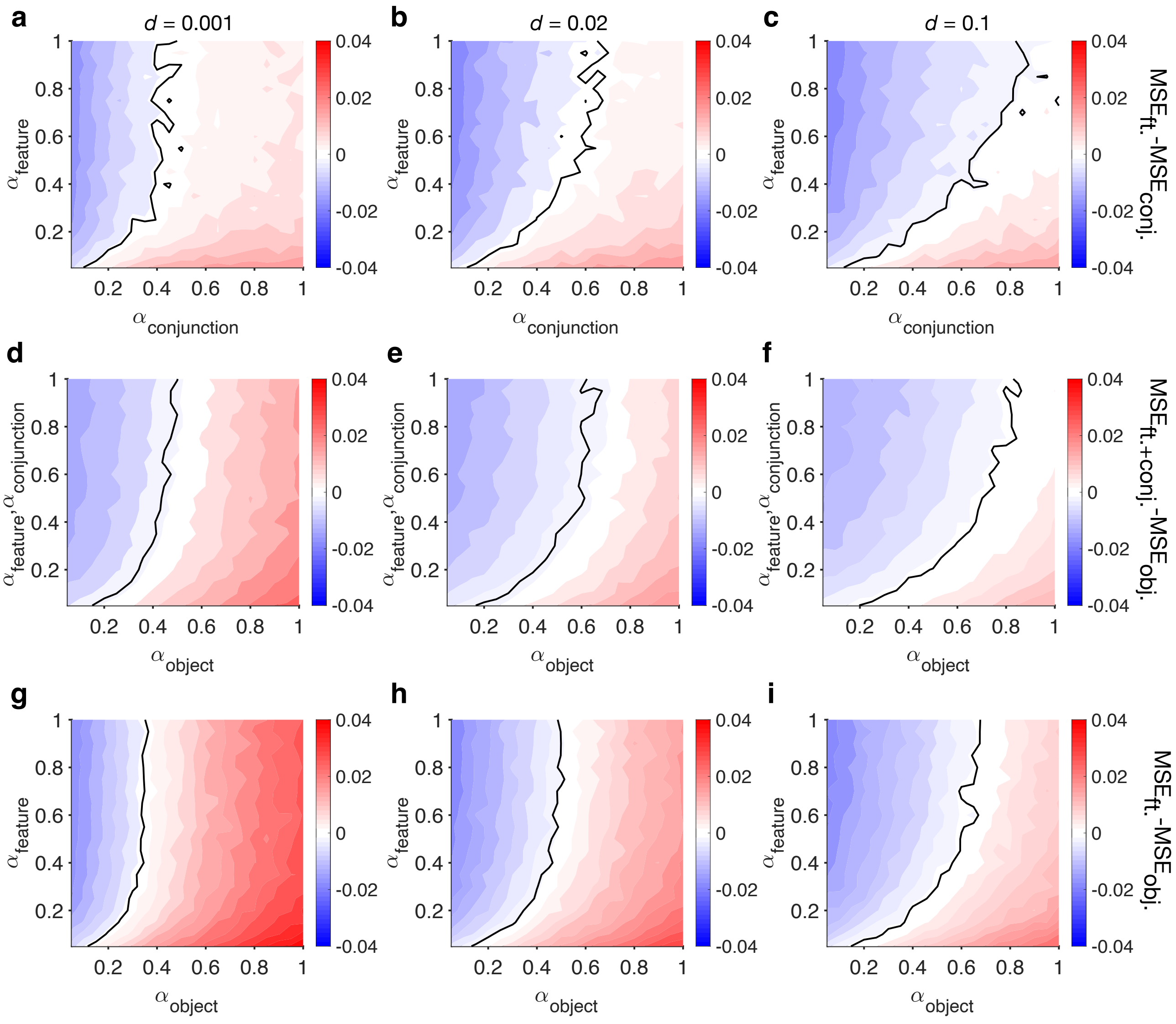

Supplementary Fig. 3. Comparison of estimation error between different learning strategies. (a–c) Plots show the difference in the average squared error (MSE) of a feature-based learner and a conjunction-based learner in the estimation of reward probabilities during the first 50 trials of the learning task. Reinforcement learning models based on feature-based, conjunction-based, mixed feature- and conjunction-based, and object-based learning were simulated using the same learning rates for rewarded and unrewarded trials ($\alpha_{\text{rew}} = \alpha_{\text{unr}}$) but different values for the decay rate (d = 0.001 (a), d = 0.02 (b), d = 0.1 (c)) for unchosen options. The black curve indicates parameter values for which the difference is equal to 0 corresponding to similar precision of feature-based and conjunction-based learners. (**d–f**) Same as (a–c) but comparing object-based and F+C_1 learners. (**g–i**) Same as (a–c) but comparing object-based and feature-based learners. Source data are provided as a Source Data file.

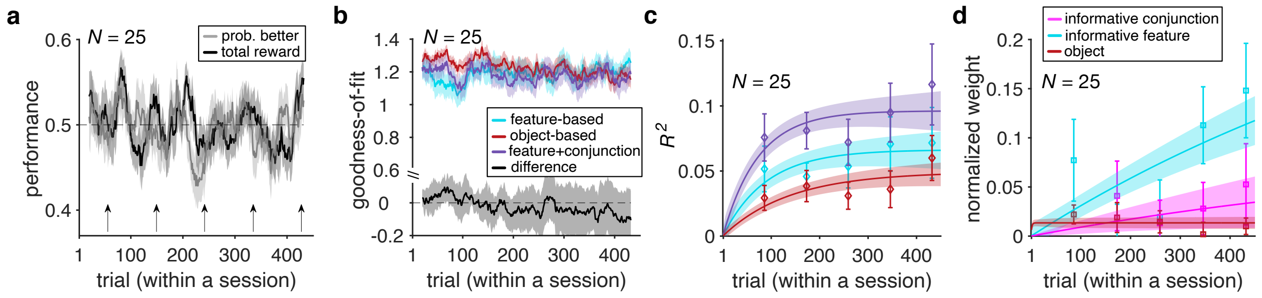

Supplementary Fig. 4. Analyses of choice behavior and estimation of excluded participants. (**a**) Time course of performance and learning during the experiment. Plotted are the total harvested reward and probability of selecting the stimulus with higher probability of reward (better option) in a given trial within a session of the experiment. The running average over time is computed using a moving box with the length of 20 trials. The shaded areas indicate +/- s.e.m., and the dashed line shows chance performance. Overall, these participants failed to learn reward probabilities associated with the options. (**b**) Plotted is the goodness-of-fit based on the average AIC per trial, AIC_p, for the feature-based model, object-based model, and the best mixed feature- and conjunction-based (F+C_1) model. The shaded areas indicate +/- s.e.m. The smaller value corresponds to a better fit. The black curve shows the difference between the goodness-of-fit for the F+C_1 and feature-based models. Throughout most of the experiment, the F+C_1 model provided a better fit for choice behavior of excluded participants, similarly to that for the participants included in the study. (**c**) The plot shows the time course of explained variance (R^2) in participants' estimates based on different GLMs. Color conventions are the same as in panel (b) with cyan, red, and purple curves representing R^2 based on feature-based, object-based, and the F+C_1 models, respectively. The solid line is the average of fitted exponential function to each participant's data, and the shaded areas indicate +/- s.e.m. of the fit. (**d**) Time course of adopted learning strategies measured by fitting participants' estimates of reward probabilities using a stepwise GLM. Plotted is the normalized weight of the informative feature, informative conjunction, and object (stimulus identity) on reward probability estimates. Error bars represent s.e.m. The solid line is the average of fitted exponential function to each participant's data, and the shaded areas indicate +/- s.e.m. of the fit. Overall, we found no evidence that the excluded participants adopted a strategy qualitatively different from the one used by the rest of the participants. Source data are provided as a Source Data file.

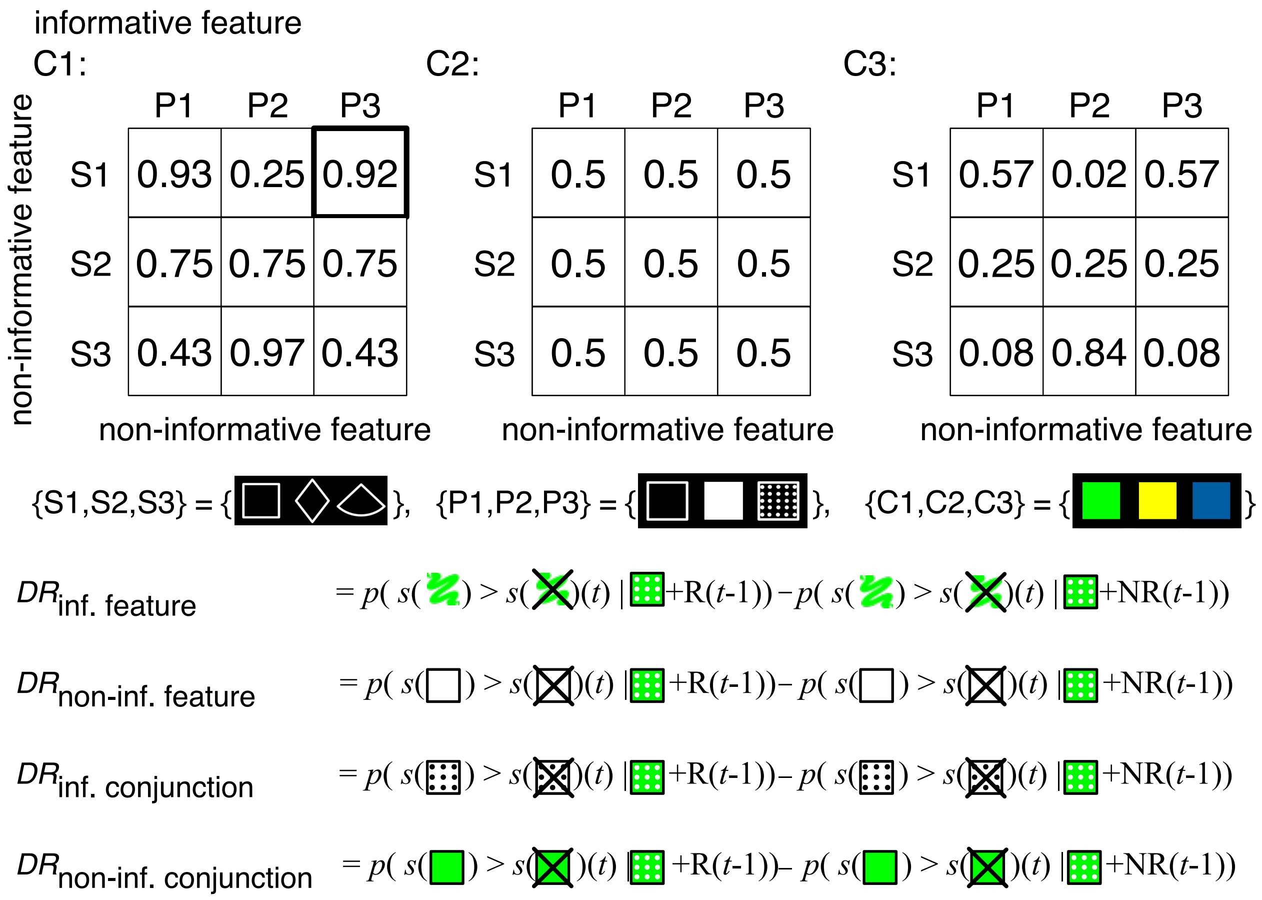

Supplementary Fig. 5. Differential response to reward feedback as a proxy for learning strategy. An example reward schedule and rules used to calculate differential responses for an example of chosen stimulus (green polka dot square) defined with three features: C1, S1, P3. Differential response for the informative or non-informative features is defined as the probability of selecting stimuli that contains only the informative feature (e.g., color in the example schedule) or non-informative features (e.g., shape and pattern) of the stimulus selected and rewarded in the previous trial minus the same probability when the previous trial was not rewarded. Similarly, differential response for informative conjunctions (e.g., conjunctions of shape and pattern in the example schedule) and non-informative conjunctions (e.g., conjunction of color and shape) is calculated for stimuli that contain the conjunction of non-informative features of the stimulus selected in the previous trial. The probability of choosing a stimuli with feature or conjunction of features X when it is presented together with a stimuli with feature or conjunction of features Y in trial t given that selection of Z was rewarded (R) and unrewarded (NR) in the previous trial is denoted as $p(s(X)\succ s(Y)(t) \mid Z\text{+R}(t\text{-}1))$ and $p(s(X)\succ s(Y)(t) \mid Z\text{+NR}(t\text{-}1))$, respectively. All these probabilities were calculated for trials where the chosen stimulus on trial t was paired with a stimulus that did not share any features or conjunction of features with the previously chosen stimulus (shown with a cross mark).

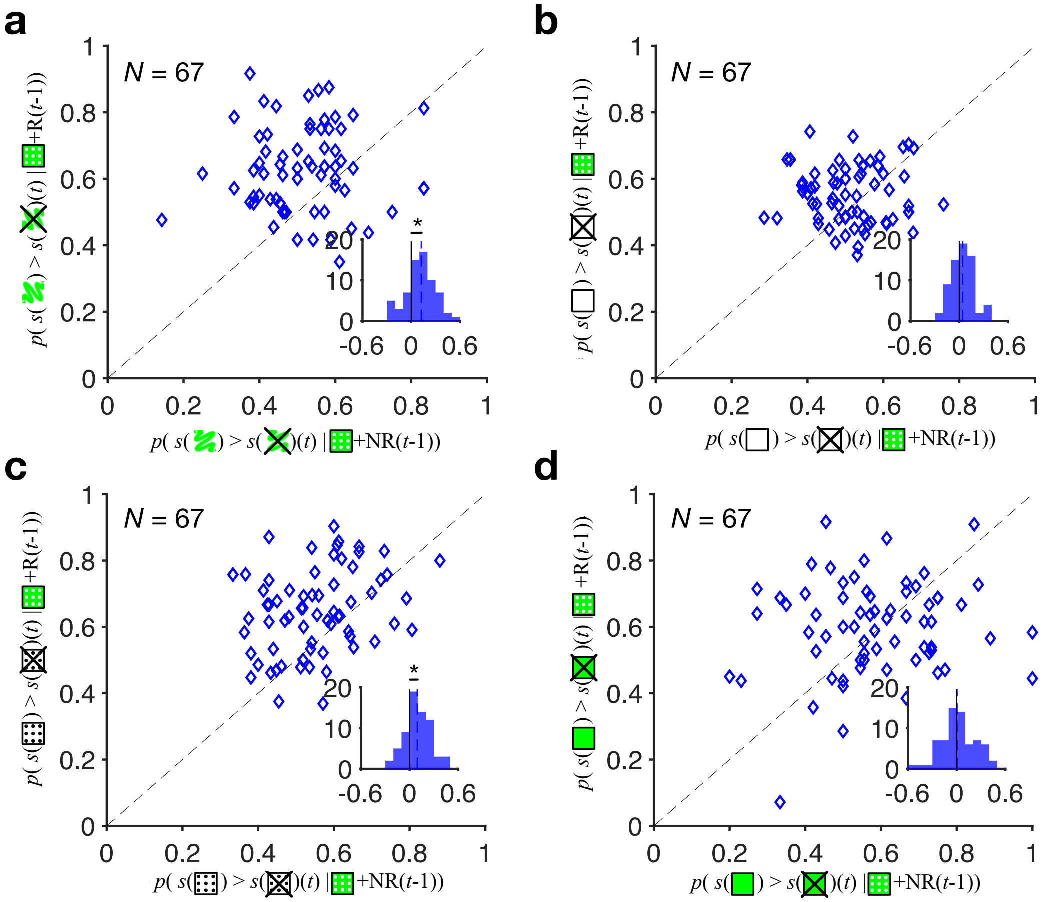

Supplementary Fig. 6. Response to reward feedback depends on the informativeness of a feature or conjunction of features in the stimulus selected in the previous trial. (**a**) Plotted is the probability of selecting stimuli that contained the informative feature of the stimulus that was selected and rewarded (R) in the previous trial versus the same probability when the previous trial was not rewarded (NR). The inset shows the histogram of the difference between these two probabilities (i.e., differential response). The dashed lines show the median values across participants, and the asterisk indicates the median is significantly different from 0 (two-sided sign-rank test; $P = 10^{-3}$). (**b**) Similar to (a) but for stimuli that contained the non-informative features of the stimulus that was selected and rewarded in the previous trial versus the same probability when the previous trial was not rewarded (two-sided sign-rank test; $P = 0.09$). (**c–d**) Similar to (a–b) but for the informative and non-informative conjunctions (two-sided sign-rank test; informative conjunction: $P = 2.7\times10^{-3}$; non-informative conjunctions: $P = 0.11$). Source data are provided as a Source Data file.

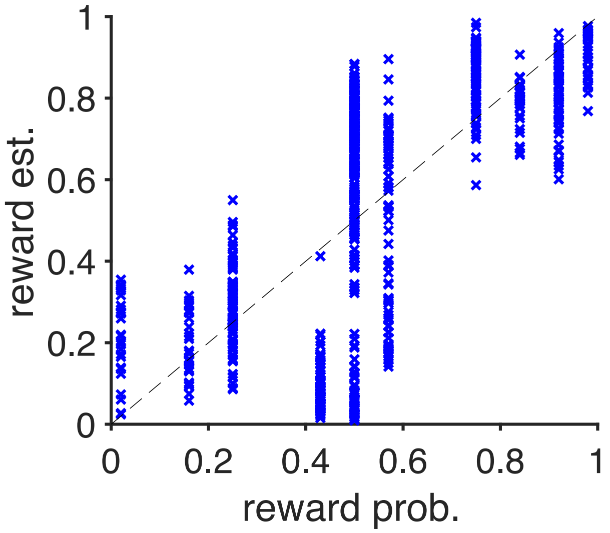

Supplementary Fig. 7. Trained networks are able to generalize to stimuli that were not used during learning. The trained RNNs performed a session of the task but with only a subset of the stimuli (a random set of 18 stimuli out of the 27 stimuli) and were tasked to predict the value for the subset of the stimuli not shown during learning (leave-out stimuli). Each point shows the predicted reward probability for a leave-out stimulus vs. its actual reward probability. The dashed line shows the identity line. Estimated reward probabilities for leave-out stimuli were significantly correlated with their actual reward probabilities (spearman correlation; $\rho = 0.77$, $P = 1.7\times10^{-7}$). Moreover, estimated reward probabilities of leave-out stimuli could deviate from the actual reward probability of these stimuli, confirming that the trained RNNs were not overfitting. Source data are provided as a Source Data file.

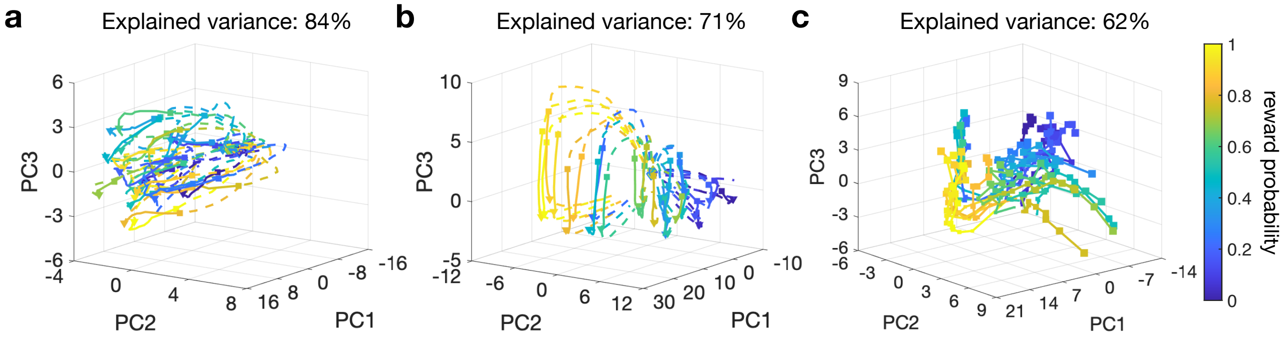

Supplementary Fig. 8. Dynamics of population activity in RNNs during the learning task. (a–b) Trajectories in the activity space formed by the first three principal components of PCA performed on the response of excitatory recurrent populations at the beginning (a) and end of each session (b). Diamonds mark stimulus onset, squares mark beginning of the choice period, and triangles mark the end of stimulus presentation. Different colors represent reward value (probability) assigned to each stimulus. (**c**) Trajectories in the activity space formed by the first three principal components of PCA performed on the response of excitatory recurrent populations during the choice period as the network learns about different stimuli. Larger markers indicate later trials within the session. Source data are provided as a Source Data file.

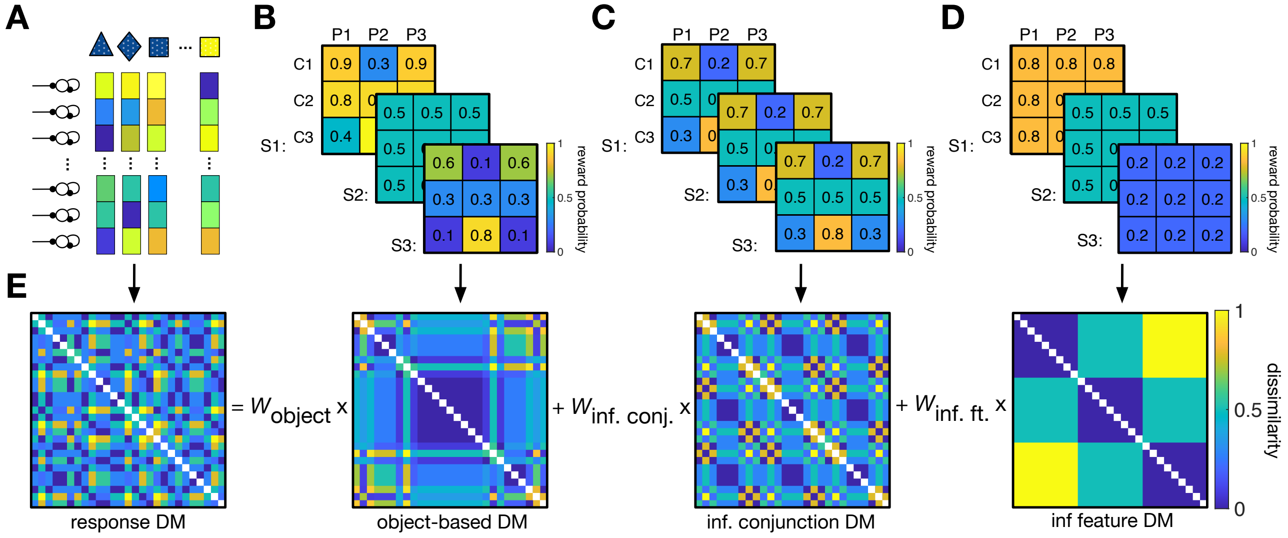

Supplementary Fig. 9. Schematic of the representational similarity analysis (RSA) used in this study. (**a**) The response dissimilarity matrix (DM) was computed as the Euclidean distance between the activity of recurrent populations in a certain population (e.g., Exc_{rr}) during the choice period. (**b–d**) The reward probability DMs were calculated as the Euclidean distance between reward probability estimates based on an object-based model (b), a model based on the conjunctions of non-informative features (i.e., the informative conjunction) (c), and a model based on the informative feature (d) for all the stimuli used in the experiment. (**e**) As the final step of RSA, a GLM is used to fit the response DM as a function of the normalized weights of the three reward probability dissimilarity matrices.

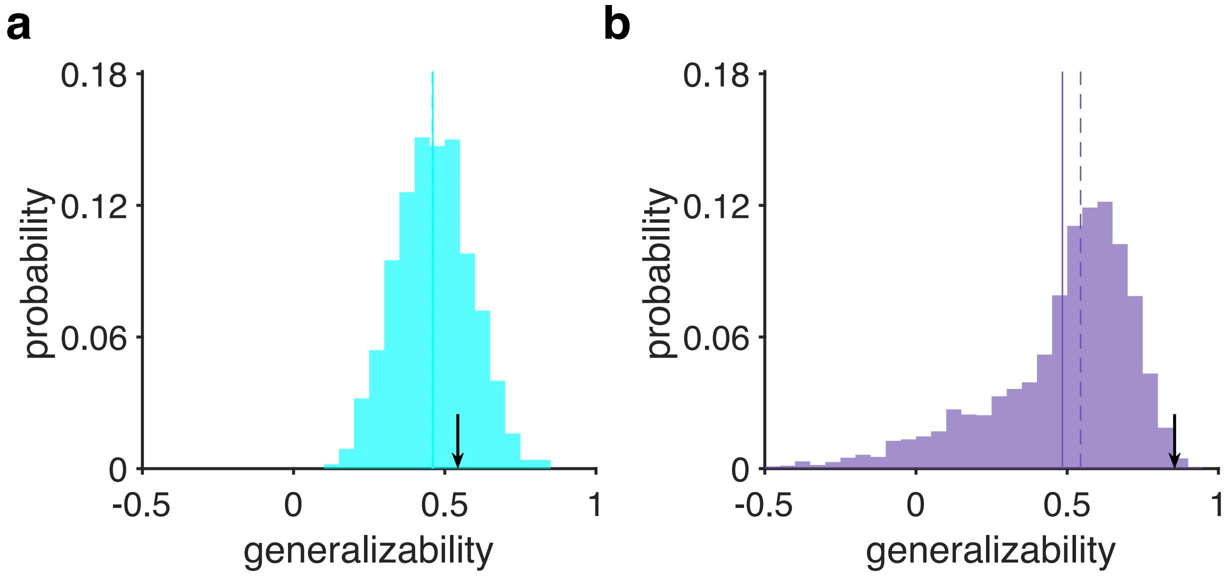

Supplementary Fig. 10. Distribution of generalizability indices in the environments used for training the RNNs. The plots show the distribution of generalizability indices calculated for the estimated reward probabilities associated with different stimuli based on their features (a) and the mixture of individual features and conjunctions (b). The dashed and solid lines show the mean and median of the distributions, respectively. The arrows show the generalizability values associated with the reward schedule used in our task. Source data are provided as a Source Data file.

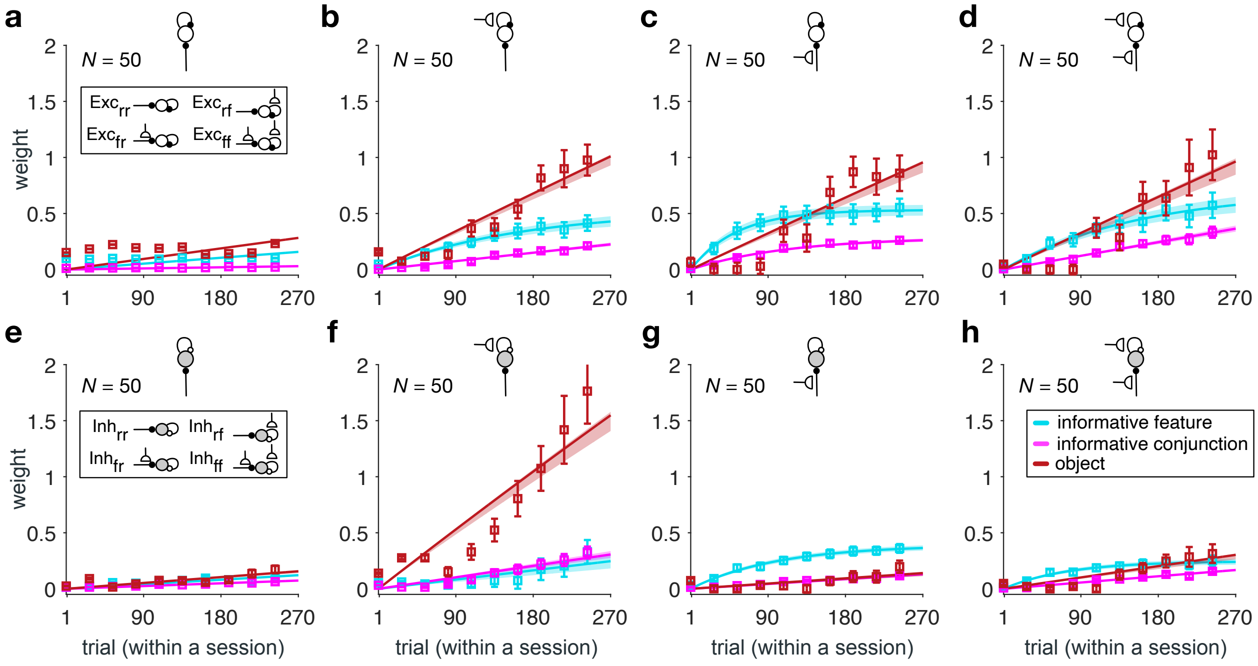

Supplementary Fig. 11. Similarity of response in different recurrent populations to reward probabilities based on different learning strategies for the RNNs in which recurrent connections from the inhibitory populations with plastic sensory inputs (Inh_{fr} and Inh_{ff}) to the excitatory populations with plastic sensory inputs (Exc_{fr} and Exc_{ff}) are lesioned. (**a**–**d**) Plotted are the estimated weights for predicting the response dissimilarity matrix of different types of recurrent populations (indicated by the inset diagrams explained in **Figure 4b**) using the dissimilarity of reward probabilities based on the informative feature, informative conjunction, and object. Error bars represent s.e.m. The solid line is the average of fitted exponential functions to RNNs' data, and the shaded areas indicate +/- s.e.m. of the fit. (**e**–**h**) Same as (a–d) but for inhibitory recurrent populations. Source data are provided as a Source Data file.

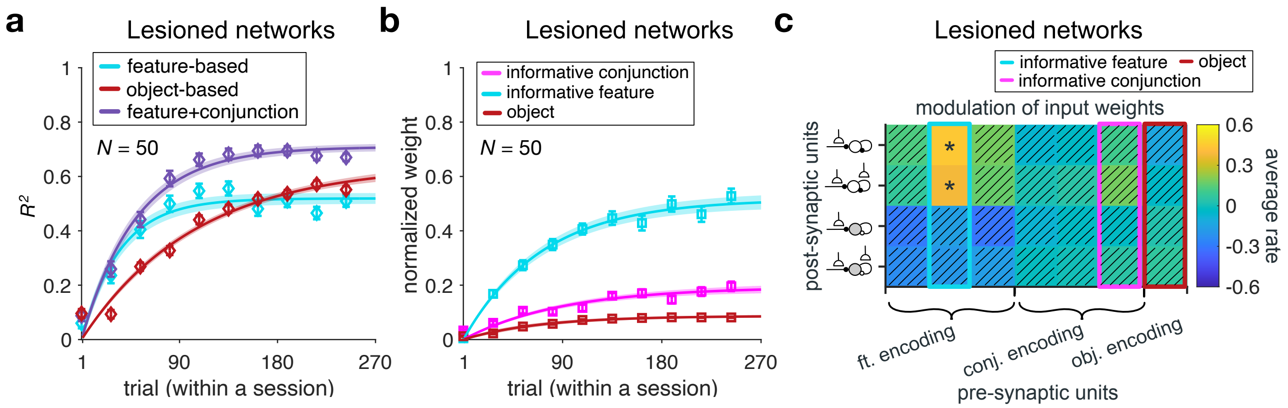

Supplementary Fig. 12. Results of lesioning connections from the excitatory populations with plastic sensory input (Exc_{fr} and Exc_{ff}) to the inhibitory populations with plastic sensory input (Inh_{fr} and Inh_{ff}) in the RNNs. (**a**) The plot shows the time course of explained variance (R^2) in RNNs' estimates based on different models. Error bars represent s.e.m. The solid line is the average of exponential fits to RNNs' data, and the shaded areas indicate +/- s.e.m. of the fit. (**b**) Time course of adopted learning strategies measured by fitting the RNNs' output. Plotted is the normalized weight of the informative feature, informative conjunction, and object (stimulus identity) on reward probability estimates ($\tau_{\text{inf. feature}} = 76.34$, $\tau_{\text{inf.conjunction}} = 91.08$). Error bars represent s.e.m. The solid line is the average of exponential fits to RNNs' data, and the shaded areas indicate +/- s.e.m. of the fit. (**c**) Plotted is the average rate of value-dependent changes in the connection weights from feature-encoding, conjunction-encoding, and object-identity encoding populations to recurrent populations with plastic sensory input during the simulation of our task. Asterisks indicate significant rates of change (two-sided sign-rank test; $P<0.05$), whereas hatched squares indicate connections with rates of change that were not significantly different from zero (two-sided sign-rank test; $P>0.05$). Highlighted rectangles in cyan, magenta, and red indicate the values for input from sensory units encoding the informative feature, the informative conjunction, and object-identity, respectively. Source data are provided as a Source Data file.

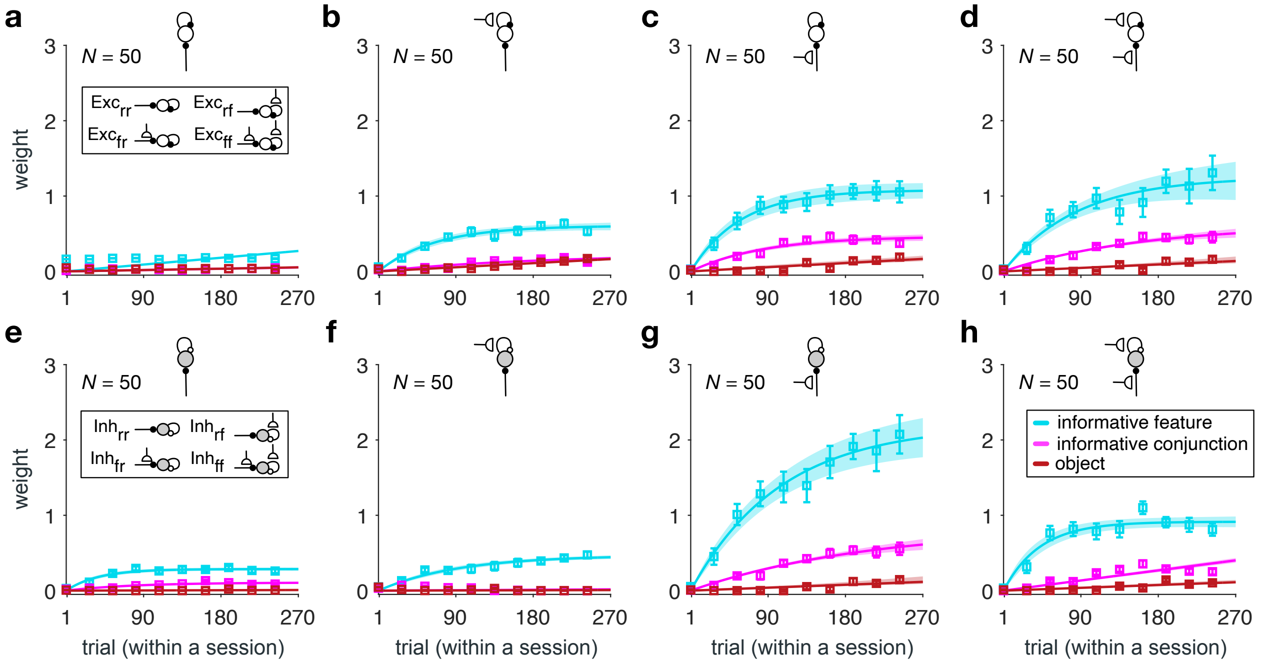

Supplementary Fig. 13. Similarity of response in different recurrent populations to reward probabilities based on different learning strategies for the RNNs in which recurrent connections from the excitatory populations with plastic sensory input (Exc$_{fr}$ and Exc$_{ff}$) to the inhibitory populations with plastic sensory input (Inh$_{fr}$ and Inh$_{ff}$) are lesioned. (**a**–**d**) Plotted are the estimated weights for predicting the response dissimilarity matrix of different types of recurrent populations (indicated by the inset diagrams explained in **Figure 4b**) using the dissimilarity of reward probabilities based on the informative feature, informative conjunction, and object. Error bars represent s.e.m. The solid line is the average of fitted exponential functions to RNNs' data and the shaded areas indicate +/- s.e.m. of the fit. (**e**–**h**) Same as (a–d) but for inhibitory recurrent populations. Source data are provided as a Source Data file.

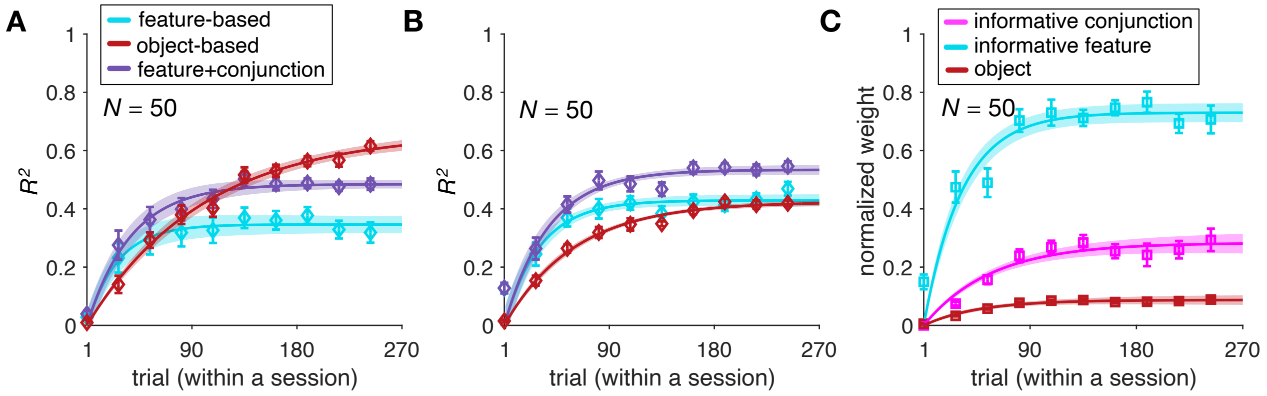

Supplementary Fig. 14. RNNs without reward-dependent plasticity in recurrent connections and feedforward neural networks (FFNNs) fail to replicate experimental results. (**a–b**) The plots show the time course of explained variance (R^2) in RNNs' estimates based on different models for the RNNs without reward-dependent plasticity in recurrent connections (a) and FFNNs (b). Error bars represent s.e.m. The solid line is the average of exponential fits to RNNs' data, and the shaded areas indicate +/- s.e.m. of the fit. (**c**) Time course of adopted learning strategies measured by fitting the RNNs' output. Plotted is the normalized weight of informative feature, the informative conjunction, and object (stimulus identity) on reward probability estimates in the FFNNs ($\tau_{\text{inf. feature}} = 37.04$, $\tau_{\text{inf. conjunction}} = 50.18$). Error bars represent s.e.m. The solid line is the average of exponential fits to RNNs' data, and the shaded areas indicate +/- s.e.m. of the fit. Source data are provided as a Source Data file.

Model	Feature-based	Mixed feature- and conjunction-based			Object-based	Mixed feature- and object-based			
		F+C_1	F+C_2	F+C_3		F_1+O	F_2+O	F_3+O	
# pars.	6	7	7	7	4	7	7	7	
-LL	254.4±5.2	253.2±5.2	259.8±4.9	251.0±5.0	274.0±2.5	252.6±5.2	271.2±5.1	271.4±5.2	Full-update
AIC	520.7±10.5	520.4±10.5	533.6±9.8	516.1±10.1	556.1±4.9	519.3±10.4	556.4±10.2	556.9±10.5	
BIC	545.1±10.5	548.8±10.5	562.1±9.8	544.5±10.1	572.4±4.9	547.8±10.4	584.9±10.2	585.4±10.5	
-LL	246.1±5.3	242.4±5.3 (P=0.06)	255.2±5.3	244.3±5.0	252.7±4.2	249.2±5.2	249.1±4.1	251.1±4.2	Chosen-update
AIC	504.1±10.6	498.8±10.6 (P=0.03)	524.3±10.6	502.6±10.1	513.4±8.4	512.0±10.5	512.2±8.2	518.2±8.2	
BIC	528.5±10.6	527.2±10.6 (P=0.04)	531.0±10.1	531.0±10.1	529.7±8.4	540.9±10.5	540.5±8.2	540.6±8.2	
# pars.	7	8	8	8	5	8	8	8	
-LL	245.5±5.2 (P=0.004)	233.5±5.1	261.8±5.3	240.8±4.9	247.6±4.0 (P=0.001)	246.1±5.3	247.4±4.2	246.9±4.3	Decay
AIC	505.1±10.4 (P=0.026)	483.1±10.3	539.7±10.7	497.7±9.8	505.2±7.9 (P=0.005)	508.2±10.7	510.8±8.4	509.7±8.6	
BIC	533.5±10.4 (P=0.034)	515.6±10.3	572.2±10.7	530.2±9.8	525.5±7.9 (P=0.01)	540.8±10.7	543.3±8.4	542.3±8.6	

Supplementary Table 1. Comparison of the goodness-of-fit measures for fitting choice data of human participants. Reported are the goodness-of-fit measures, negative log likelihood (-LL), Akaike information criterion (AIC), and Bayesian information criterion (BIC) averaged over all participants (mean±s.e.m.) for different groups of models. The mixed model providing the best fit (F+C_1) and its object-based and feature-based counterparts are highlighted in green and orange, respectively. The values in parentheses for the feature-based with decay model and the object-based with decay model indicate comparison with the F+C_1 with decay model, using a two-sided, sign-rank test. The values in parentheses for the chosen-update F+C_1 model indicate comparison with the F+C_1 model with decay using two-sided, sign-rank test. Source data are provided as a Source Data file.

Model	Feature-based	Mixed feature- and conjunction-based			Object-based	Mixed feature- and object-based			
		$F+C_1$	$F+C_2$	$F+C_3$		F_1+O	F_2+O	F_3+O	
# pars.	5	6	6	6	3	6	6	6	
-LL	167.5±3.4	163.4±3.4	168.8±3.3	169.1±3.3	173.0±3.0	169.7±3.5	171.7±3.6	172.5±3.7	Full-update
AIC	345.1±6.8	337.9±6.9	349.5±6.7	350.9±6.6	352.0±6.2	346.3±6.9	343.4±7.2	351.0±7.5	
BIC	363.1±6.8	360.6±69	371.1±6.7	372.4±6.6	362.8±6.2	368.9±6.9	371.0±7.2	372.6±7.5	
-LL	167.1±3.4	162.9±3.4	167.9±3.2	168.3±3.0	172.6±3.0	169±3.7	170.4±3.6	172.0±3.6	Chosen-update
AIC	344.3±6.7	336.9±6.8	347.7±6.5	348.7±6.2	351.2±6.0	345.9±7.4	346.8±7.2	350.1±7.1	
BIC	362.3±6.7	359.6±6.8	369.3±6.5	370.3±6.2	362.0±6.0	367.5±7.4	368.4±7.2	371.7±7.1	
# pars.	6	7	7	7	4	7	7	7	
-LL	167.2±3.5 (*P*=0.007)	161.7±3.4	167.3±3.0	170.0±3.0	171.8±2.8 (*P*=0.003)	166.8±3.6	167.4±3.6	167.9±3.6	Decay
AIC	346.4±6.9 (*P*=0.022)	337.4±6.7	348.7±6.2	354.0±6.0	351.7±5.6 (*P*=0.003)	347.7±7.2	348.9±7.2	349.9±7.3	
BIC	368.0±6.9 (*P*=0.039)	362.6±6.7	373.9±6.2	379.2±6.0	366.0±5.6 (*P*=0.004)	372.9±7.2	374.0±7.2	375.0±7.3	

Supplementary Table 2. Comparison of the goodness-of-fit measures for fitting choice data generated by the trained RNNs. Reported are the goodness-of-fit measures, negative log likelihood (-LL), Akaike information criterion (AIC), and Bayesian information criterion (BIC) averaged over all trained RNNs (mean±s.e.m.). The model providing the best fit ($F+C_1$) and its object-based and feature-based counterparts are highlighted in green and orange, respectively. The values in parentheses for the feature-based with decay model and the object-based with decay model indicate comparison with the $F+C_1$ with decay model, using a two-sided, sign-rank test. Source data are provided as a Source Data file.
