# Supplementary material for: Computational mechanisms of distributed value representations and mixed learning strategies
Source: Nat Commun. 2021 Dec 10;12:7191. doi: 10.1038/s41467-021-27413-2 (PMC8664930; doi:10.1038/s41467-021-27413-2)
Supplement: Supplementary file 3 — Reporting summary [file 41467_2021_27413_MOESM3_ESM.pdf]

nature research

Corresponding author(s): Alireza Soltani, Shiva Farashahi

Last updated by author(s): Oct 25, 2021

Reporting Summary

Nature Research wishes to improve the reproducibility of the work that we publish. This form provides structure for consistency and transparency in reporting. For further information on Nature Research policies, see our Editorial Policies and the Editorial Policy Checklist.

Statistics

For all statistical analyses, confirm that the following items are present in the figure legend, table legend, main text, or Methods section.

n/a	Confirmed	
☐	☒	The exact sample size (*n*) for each experimental group/condition, given as a discrete number and unit of measurement
☐	☒	A statement on whether measurements were taken from distinct samples or whether the same sample was measured repeatedly
☐	☒	The statistical test(s) used AND whether they are one- or two-sided<br>*Only common tests should be described solely by name; describe more complex techniques in the Methods section.*
☒	☐	A description of all covariates tested
☐	☒	A description of any assumptions or corrections, such as tests of normality and adjustment for multiple comparisons
☐	☒	A full description of the statistical parameters including central tendency (e.g. means) or other basic estimates (e.g. regression coefficient) AND variation (e.g. standard deviation) or associated estimates of uncertainty (e.g. confidence intervals)
☐	☒	For null hypothesis testing, the test statistic (e.g. *F*, *t*, *r*) with confidence intervals, effect sizes, degrees of freedom and *P* value noted<br>*Give P values as exact values whenever suitable.*
☒	☐	For Bayesian analysis, information on the choice of priors and Markov chain Monte Carlo settings
☒	☐	For hierarchical and complex designs, identification of the appropriate level for tests and full reporting of outcomes
☐	☒	Estimates of effect sizes (e.g. Cohen's *d*, Pearson's *r*), indicating how they were calculated

Our web collection on statistics for biologists contains articles on many of the points above.

Software and code

Policy information about availability of computer code

Data collection	All data was collected using custom code written in Matlab with Psychtoolbox Version 3 extensions. Details are specified in Methods.
Data analysis	All behavioral analyses and model fitting were done using custom code written in MATLAB 2018a. RNN simulations were implemented using the Tensorflow package in Python 3.7 environment. Details of the different analyses are specified in the main text and Methods.The source code to reproduce the results of this study has been deposited in a public repository accessible at https://doi.org/10.5281/zenodo.5594684.

For manuscripts utilizing custom algorithms or software that are central to the research but not yet described in published literature, software must be made available to editors and reviewers. We strongly encourage code deposition in a community repository (e.g. GitHub). See the Nature Research guidelines for submitting code & software for further information.

Data

Policy information about availability of data

All manuscripts must include a data availability statement. This statement should provide the following information, where applicable:

- Accession codes, unique identifiers, or web links for publicly available datasets
- A list of figures that have associated raw data
- A description of any restrictions on data availability

The raw and processed data generated in this study have been deposited in a GitHub repository accessible https://doi.org/10.5281/zenodo.5594684.

Field-specific reporting

Please select the one below that is the best fit for your research. If you are not sure, read the appropriate sections before making your selection.

- [x] Life sciences
- [ ] Behavioural & social sciences
- [ ] Ecological, evolutionary & environmental sciences

For a reference copy of the document with all sections, see nature.com/documents/nr-reporting-summary-flat.pdf

Life sciences study design

All studies must disclose on these points even when the disclosure is negative.

Sample size	We did not perform sample size calculations. The sample size for behavioral data are over-powered compared to similar types of studies (for example: Niv et al., JN 2015; Farashahi et al, Nat Comm, 2017).
Data exclusions	Participants whose performance was not significantly different from chance were excluded from the main analyses. These criterion was established prior to data collection. Details are specified in Methods. Nonetheless, we show the results of data analyses on excluded participants in the Supplementary Information.
Replication	We collected a large sample to identify adoption of different types of learning strategies among participants. Due to COVID-19 Pandemic, replication of such a large dataset was not feasible.
Randomization	All subjects included were studied on the same task, hence no randomization of subjects was required.
Blinding	No group allocation was performed because all subjects behavior were studied on the same tasks.

Reporting for specific materials, systems and methods

We require information from authors about some types of materials, experimental systems and methods used in many studies. Here, indicate whether each material, system or method listed is relevant to your study. If you are not sure if a list item applies to your research, read the appropriate section before selecting a response.

Materials & experimental systems

n/a	Involved in the study	
x		Antibodies
x		Eukaryotic cell lines
x		Palaeontology and archaeology
x		Animals and other organisms
	x	Human research participants
x		Clinical data
x		Dual use research of concern

Methods

n/a	Involved in the study	
x		ChIP-seq
x		Flow cytometry
x		MRI-based neuroimaging

Human research participants

Policy information about studies involving human research participants

Population characteristics	Participants were recruited from the Dartmouth College student population (ages 18–22 years). In total, 92 subjects were recruited (66 females) to perform the task. Details are specified in the Methods.
Recruitment	Participants were recruited through Department of Psychological and Brain Sciences experiment scheduling system at Dartmouth College. Subjects were compensated with a combination of money and "T-points," which are extra credit points for classes within the Department of Psychological and Brain Sciences at Dartmouth College. All experimental procedures were approved by the Dartmouth College Institutional Review Board, and informed consent was obtained from all participants before the experiment.
Ethics oversight	All experimental procedures in humans were approved by the Dartmouth College Institutional Review Board.

Note that full information on the approval of the study protocol must also be provided in the manuscript.
